# Hyperspectral analysis for perioperative perfusion monitoring—a clinical feasibility study on free and pedicled flaps

**DOI:** 10.1007/s00784-020-03382-6

**Published:** 2020-06-15

**Authors:** D. G. E. Thiem, R. W. Frick, E. Goetze, M. Gielisch, B. Al-Nawas, P. W. Kämmerer

**Affiliations:** 1 Department of Oral and Maxillofacial Surgery, Facial Plastic Surgery, University Medical Centre Mainz, Augustusplatz 2, 55131 Mainz, Germany; 2 Department of Oral and Maxillofacial Surgery, School of Dentistry, Kyung Hee University, Seoul, Korea

**Keywords:** Hyperspectral imaging, Flap perfusion, Flap monitoring, Non-invasive, Contactless

## Abstract

**Objectives:**

In reconstructive surgery, flap monitoring is crucial for early identification of perfusion problems. Using hyperspectral imaging (HSI), this clinical study aimed to develop a non-invasive, objective approach for perfusion monitoring of free and pedicled flaps.

**Material and methods:**

HSI of 22 free (FF) and 8 pedicled flaps (PF) in 30 patients was recorded over time. Parameters assessed were tissue oxygenation/superficial perfusion (0–1 mm) (StO_2_ (0–100%)), near-infrared perfusion/deep perfusion (0–4 mm) (NIR (0–100)), distribution of haemoglobin (THI (0–100)), and water (TWI (0–100)). Measurements up to 72 h were correlated to clinical assessment.

**Results:**

Directly after flap inset, mean StO_2_ was significantly higher in FF (70.3 ± 13.6%) compared with PF 56.2 ± 14.2% (*p* = 0.05), whereas NIR, THI, and TWI were similar (NIR_*p* = 0.82, THI_*p* = 0.97, TWI_*p* = 0.27). After 24 h, StO_2_, NIR, THI, and TWI did not differ between FF and PF. After 48 h, StO_2_, NIR, and TWI did not differ between FF and PF whereas THI was significantly increased in FF compared with PF(*p* = 0.001). In three FF, perfusion decreased clinically and in HSI, 36^(1)^, 40^(2)^, 5^(3^), and 61^(3)^ h after flap inset which was followed by prompt intervention.

**Conclusions:**

StO_2_ < 40%, NIR < 25/100, and THI < 40/100 indicated arterial occlusion, whereas venous problems revealed an increase of THI. In comparison with FF, perfusion parameters of PF were decreased after flap transfer but remained similar to FF later on.

**Clinical relevance:**

HSI provides objective and non-invasive perfusion monitoring after flap transplantation in accordance to the clinical situation. With HSI, signs of deterioration can be detected hours before clinical diagnosis.

**Electronic supplementary material:**

The online version of this article (10.1007/s00784-020-03382-6) contains supplementary material, which is available to authorized users.

## Introduction

Free flap and pedicled flap survival relies on adequate tissue perfusion. As free flaps are anastomosed to suitable donor vessels, pedicled flaps become translocated to the recipient region while staying constantly connected to the supplying vessels. Flap failures may be attributed to arterial or venous occlusion due to thrombosis, external compression, vessel kinking, or the formation of haematoma [1]. In this context, evidence indicates that timely re-exploration significantly increases the rate of compromised flap salvage; thus, close postoperative monitoring is necessary in order to detect early signs of deterioration and to revise if necessary [2, 3]. The risk of a total loss following free microvascular tissue transfer is approximately 2–6%, mainly due to thrombotic events in the flow area of the microanastomosis [4, 5]. A wealth of objective and subjective monitoring procedures have been reported in the past, but the current gold standard in monitoring flap perfusion is still based on a subjective clinical bedside assessment [1, 6]. Here, Chubb et al. reviewed 1140 clinically monitored cases with overall 94 surgical revisions, showing a false-positive rate of 0.4% and an overall flap salvage rate of 62.8% [7]. Though an expert-investigator cannot be around at all time, another valid monitoring should be guaranteed after surgery. Hence, there is an increasing demand for an objective, reliable, and investigator-independent method to assess flap perfusion. Hyperspectral imaging (HSI) could be suitable for clinical use as it is a non-contact, non-ionising, and non-invasive monitoring technique that provides objective, reproducible, precise, and relevant information about physiological parameters in different medical fields of application like tissue perfusion measurements and wound assessment [8–10]. HSI processes the optical properties of a large area in a wavelength range from visual light (approx. 380–740 nm) to near infrared (750–1000 nm; NIR), acquiring a 3D data set (“hypercube”) [10]. The TIVITA™ Tissue System software is trained to detect and measure haemoglobin with its derivatives oxyhaemoglobin (O_2_Hb), deoxyhaemoglobin (HHb), and water to analyse the cutaneous and subcutaneous oxygenation pattern (StO_2_ and NIR Perfusion Index) [10]. Therefore, this study aimed to investigate the primary question whether the use of hyperspectral imaging is feasible for objective and reproducible monitoring of flap perfusion in patients following reconstruction by free and pedicled flaps in comparison with clinical bedside assessment. Moreover, this study aimed to provide threshold values by means of an interpretation aid for clinicians to simplify the postoperative flap assessment.

## Material and methods

### Patients

In this prospective, non-randomised, clinical cohort study, patients with either free or pedicled flaps for reconstruction in the oro-maxillofacial area were included. The study was approved by the local ethic committee of Rhineland-Palate (registration number: 2019-14312) and was conducted in accordance with the protocol and in compliance with the moral, ethical, and scientific principles governing clinical research as set out in the Declaration of Helsinki of 1975 as revised in 1983. As the oral cavity is not accessible via HSI in the conscious patient at all sides due to light extinction, only free and pedicled flaps in the anterior lateral sides of the upper and lower jaws as well as flaps with an extraoral skin island were included*.* Exclusion criteria were cases without periodic measurements at several intervals following the flap inset.

### HSI imaging

In this study, a new hyperspectral camera system (TIVITA™ Tissue System, Diaspective Vision GmbH, Pepelow, Germany) was used. The HSI sensors generate a three-dimensional (3D) data cube, where the spatial information is contained in the first two dimensions (resolution: 0.1 mm/pixel at 30-cm distance), while the third dimension includes the spectral information (resolution, 5 nm). The light measured includes the range from 500 (visible) to 1000 nm (near-infrared), whereby the light source is arranged directly around the camera lens and consists of six halogen spotlights providing 20 W each (Table 5). Briefly, HSI is based on the assessment of contiguous spectra (i.e. light of different wavelengths) individually re-emitted by molecules, whereby the molecule-specific re-emitted wave spectrum is generated on the basis of the light spectrum of the halogen spotlights initially emitted for examination. These physico-chemical raw data are then processed by computerised algorithms, specific for the respective molecule of interest (hyperspectral signatures), particularly haemoglobin, oxygenized haemoglobin, and water. After HSI, images are recorded over 10 s, additional 8 s are needed to compute a RGD (red, green, and blue) truecolour image, and additional four pseudo-colour images, representing the physiologic parameters: tissue oxygenation/superficial perfusion (approximately 0–1 mm depth) (StO_2_ (0–100%)), near-infrared perfusion index/deep perfusion (0–4 mm depth) (NIR as arbitrary units (0–100)), and distribution of haemoglobin (THI as arbitrary units (0–100)) and water (Tissue Water Index (TWI) as arbitrary units (0–100)) [11]. Haemoglobin plays a central role in HSI perfusion analysis and the differentiation between its oxygenated and deoxygenated form. This is due to the different absorption maxima of the two forms at different wavelengths. While oxygenated haemoglobin shows a double peak in the wavelength range between 500 and 600 nm and deoxygenated haemoglobin shows a single peak, both differ particularly at 760 nm. Since the absorbance of haemoglobin in the range from 570 to 590 nm is high, electromagnetic radiation of a shorter wavelength shows a lower penetration depth in the tissue; thus, microcirculation is detected at a depth of up to 1 mm. StO_2_ reflects the percentage of haemoglobin-oxygen-saturation in the capillary area of the tissue microcirculation, records arterial and venous blood, and displays changes in oxygen supply and consumption directly on site in the tissue. Thus, StO_2_ represents the tissue oxygen saturation, which is mainly based on the blood volume in the venous part (75%) of the microcirculation and its oxygen saturation after delivery of oxygen to the tissue. Uniform standard or limit values for tissue oxygen saturation do not yet exist, although scientific studies to establish uniform limit values are still underway. The tissue oxygen saturation values of healthy volunteers are typically 50–70% [12]. The parameter NIR perfusion (near-infrared) describes the quality of blood flow, which is determined by the relative oxygen saturation of the haemoglobin and the relative haemoglobin content in the microcircular system in deeper tissue layers. The penetration depth can be 4 to 6 mm. This parameter can be used to detect undersupplied areas of tissue in deeper layers. The colour scale ranges from red (high perfusion) to blue (low perfusion). THI (Tissue Haemoglobin Index) describes the existing haemoglobin distribution in the superficial microcircular system of the tissue area under consideration. This is an index value and not an absolute value. With this parameter, it is possible to detect arterial supply or venous outflow problems. The colour scale ranges from red (high haemoglobin content) to blue (low haemoglobin content). As an index value, TWI describes the relative water content in the considered tissue area. In general, the measured values alone are not meaningful, so a consideration of combinations (THI and NIR or THI and StO_2_) is necessary. The measured oxygen saturation is the percentage of oxygen bound to haemoglobin. This is important for the determination of tissue hypoxia, since the amount of dissolved oxygen in the tissue can be determined by measuring oxygen saturation. This is due to the fact that the oxygen binding curve relates oxygen saturation to a certain amount of dissolved oxygen. If one wants to know how much oxygen is absolutely available, one needs the blood flow in addition to the oxygen saturation. Only then can the absolute amount of inflow (which is determined by the blood flow and the arterial oxygen saturation) and absolute amount of outflow (which is determined by the blood flow and the capillary-venous oxygen saturation) be used to determine the amount of oxygen delivered to the tissue. Combinations of values with the possibility of drawing conclusions about tissue supply are as follows:

- High THI and low StO_2_: venous congestion
- Low THI and low StO_2_: arterial occlusion
- Low THI and high StO_2_: following anastomosis
- Low StO_2_ and high NIR: deep tissue supply is given whereas superficial layers are undersupplied
- High StO_2_ and low NIR: critical situation as superficial supply can clinically hide saturation problems in deeper tissue layers

For accurate and reproducible measurements, the standard measuring distance was 50 cm, represented by two separate indicator light points (red and green) in an overlapped position. Measurement time points were as follows, indicating the time after the inset of the flap: t1 = directly after inset (baseline), t2 = 0–1 h, t3 = 4–8 h, t4 = 8–12 h, t5 = 12–24 h, t6 = 24–48 h, t7 = > 48 h. In the case of stalked grafts, the regularity of the measurements in the time interval t3 proved to be inconsistent, so that this time interval was not taken into account for the stalked grafts. The most likely explanation for this is the non-standardised monitoring of pedicled flaps, which led to missing measurements especially during the evening hours and at night. For quantification of StO_2_, NIR, THI, and TWI, the camera-specific software package (TIVITA™ Suite) was used in accordance to the literature [10]. In brief, at least three circularly shaped regions of interest (ROI), each with a diameter of 5 mm, were manually positioned in the recorded area/field of interest. The circular region of interests are areas that contain the mean value of the spectral and spatial information contained therein per pixel. If the diameter of the ROIs were too big, the number of mean real areas on the graft surface to be examined would be automatically reduced, and the risk of charging for a less perfused area when including hyperperfused areas would increase. The diameter of 5 mm has proven to be suitable for placing at least three ROIs on the flap surface.

**Fig. 1:**
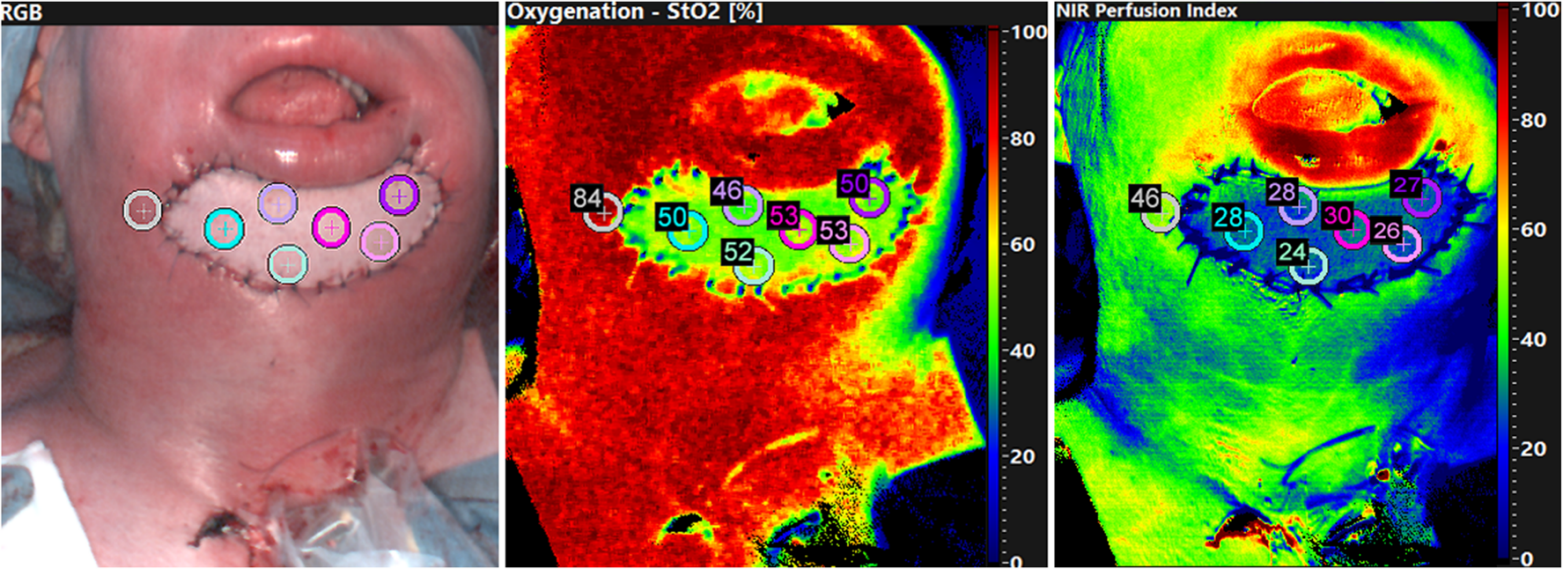
Intraoperative hyperspectral imaging directly after flap inset (t1), showing primary reconstruction after tumour resection (t4 oral squamous cell carcinoma of the anterior mandibular gingiva with bone involvement) using ALT free flap (**a**–**c**). **a** RGB (red-green-blue) true colour image with seven coloured circles labelling the different regions of interest (ROIs). The grey circles serve as reference in the adjoining area. HSI analysis of StO_2_ (**b**) and NIR perfusion index (**c**) shows the different ROIs within the flap area and the adjacent reference-ROI (grey circle). The numbers indicate the ROIs’ value in accordance to the colour scale bar on the right edge

Hence, the number of ROIs was assessed according to the flap size. ROIs were placed at different positions, whereby its number was in dependency to the flaps’ surface geometry (Fig. 1a–c) with ROIs along the border and at least one ROI in the flaps’ centre whenever possible to achieve a homogeneous distribution of the measured area. For calculation, one ROI served as reference point and was located in the adjacent normal tissue. The clinical bedside examination took place before the hyperspectral analysis, so that the examiner was not influenced by the results of the HSI. Clinical evaluation criteria for flap perfusion were flap colour (pale = ischemia; livid = venous congestion; or rosy = normal perfusion), re-capillarisation time (fast ≤ 1.5 (venous congestion); slow ≥ 3.0 s (reduced arterial blood flow); normal 1.5 to 3 s), and subjective temperature (warm versus cold). In the case of transplants with impaired perfusion, the clinical assessment of the respective case was presented separately. For all other transplants, the clinical assessment was not presented.

### Statistics

Raw data sets were saved in Excel® sheets (Microsoft Corporation, Redmond, USA) and subsequently transferred into SPSS Statistics® (version 23.0.0.2, MacOS X; SPSS Inc., IBM Corporation, Armonk, NY, USA). Data were expressed as median (MD), mean (m), standard deviation (SD ±), minimum (min), maximum (max), and standard error of the mean (SEM). Normal distribution was checked using a non-parametric Kolmogorov-Smirnov test (KS test), and results were analysed for statistical significance by the use of analysis of variance (ANOVA^(#)^), unpaired non-parametric Mann-Whitney *U* tests = ^($)^, Wilcoxon-Whitney tests = ^(§)^, and Students’ *t* test = ^(^*^)^. Considering the case number, all analyses are of explorative nature and *p* values ≤ 0.05 were termed significant for descriptive reasons only. For a proof of principle study, sample size calculation is not practicable; thus, this study is in accordance to other published proof of concept works dealing with similar group sizes [13, 14]. In the context of the sensitivity determination, the cases in which there was a decrease in perfusion parameters (StO_2_ and NRI perfusion/increase in THI) prior to flap revision were classified as correctly positive. Since there were no false-negative cases in the cases shown, the test quality must be understood as descriptive. Line charts were used for illustration purposes.

## Results

### Patients

In this study, perfusion monitoring was performed on 25 free and 8 pedicled grafts in 33 patients intra- and postoperatively using HSI. In three cases with free flaps, a revision had to be carried out so that these were listed as an extra group with separate evaluation und were therefore not included in the main statistics. Free flaps included were radial forearm flap RF-f (*n* = 12), osteocutaneous fibula flap OF-f (*n* = 4), anterolateral tight flap as perforator flaps with two perforators ALT-f (*n* = 3), and osteocutaneous scapular flap OS-f (*n* = 3), and pedicled flaps were pectoralis major flap PM-f (*n* = 3), latissimus dorsi flap LD-f (*n* = 2), nasolabial flap NL-f (*n* = 1), and large-scale scalp rotation flap LSS-f (*n* = 2).

### Comparison between free and pedicled flaps

#### T1 (baseline)

Directly after the inset of the flap, mean StO_2_ was significantly higher in FF (70.3 ± 13.6%) when compared with PF (56.2 ± 14.2%) (*p* = 0.05^(^*^)^). In contrast, mean values of NIR perfusion index (*p* = 0.82^(^*^)^), THI (*p* = 0.97^($)^), and TWI (*p* = 0.27^($)^) appeared to be similar for free and pedicled flaps (Table 1).

**Table 1:** Measured HSI parameters from t1 to t7 for free and pedicled flaps. Parameters with statistical significance (*p* ≤ 0.05) among groups are marked by two asterisks ^(^**^)^. “na” stands for not available. Significant differences between two measurement intervals within a group are marked by subscript letters, whereby the respective letters define a time interval (t1 = a, t2 = b, t3 = c, t4 = d, t5 = e, t6 = f, t7 = g)

		t1_a_ (baseline)				t2_b_ (0–1 h)				t3_c_ (4–8 h)				t4_d_ (8–12 h)				t5_e_ (12–24 h)				t6_f_ (24–48 h)				t7_g_ (> 48 h)			
		StO_2_ (%)	NIR	THI	TWI	StO_2_ (%)	NIR	THI	TWI	StO_2_ (%)	NIR	THI	TWI	StO_2_ (%)	NIR	THI	TWI	StO_2_ (%)	NIR	THI	TWI	StO_2_ (%)	NIR	THI	TWI	StO_2_ (%)	NIR	THI	TWI
Free flaps	Mean (m)	*70.3*_c,d,e,f,g_**	34.5	47.5	35.5_e,f,g_	73.1_c,d,e,f,g_	48.9_e_	27.6_d_	41.5	37.9_a,b,e,g_	35.6	41.5	44.0_g_	39.2_a,b,g_	33.1	59.4_b,f_	38.0_g_	46.8_a,b,c_	29.6_b,g_	52.7	43.3_a,g_	43.9_a,b_	33.8	43.0_d_	47.5_a_	48.2_a,b,c,d_	37.5_e_	53.7**	48.9_a,c,d,e_
	SD ±	13.6	14.8	26.6	9.5	0.84	10.1	16.8	13.4	15.7	13.8	33.3	15.8	18.0	14.1	21.5	17.9	12.6	9.3	14.5	12.6	11.2	10.5	23.1	8.3	16.4	15.7	16.7	10.2
	Median (MD)	72.1	33.7	46.4	35.6	73.1	48.8	27.6	41.4	41.0	37.8	43.0	46.1	43.6	31.6	54.0	46.0	49.2	31.6	51.3	43.7	43.2	37.0	45.8	47.0	55.5	34.7	57.4	49.1
	Min	47.7	1.3	2.8	11.5	72.5	41.7	15.7	32.0	72.5	7.0	1.0	0.0	11.0	11.0	35.0	2.0	8.0	6.0	25.8	6.0	17.5	0.0	3.6	31.0	19.8	1.5	16.0	32.3
	Max	89.9	62.0	89.5	50.2	73.7	56.0	39.5	50.9	73.7	53.2	100	58.0	71.0	60.0	98.0	56.8	68.0	45.0	98.0	68.3	64.3	56.0	93.0	70.7	69.3	76.0	91.7	69.7
	SEM	3.0	3.3	5.9	2.1	0.6	7.1	11.9	9.4	3.7	3.3	7.8	3.7	4.6	3.6	5.5	4.6	2.5	1.8	2.8	2.5	1.6	1.5	3.3	1.2	2.2	2.1	2.2	1.4
Pedicled flaps	Mean (m)	*56.2*_d_**	35.8	50.3	40.4	69.1_d,e,f_	38.6	47.1	42.5	na	na	na	na	35.9_a,b,f,g_	27.6_f_	58.2_g_	36.3	48.4_b_	31.9_b_	50.3	42.3	48.4_b,g_	38.3_d_	37.5	47.7	56.9_d,f_	34.9	*29.0*_d_**	47.4
	SD ±	14.2	10.2	28.0	8.7	15.8	7.1	19.5	1.8					5.4	8.1	18.2	10.8	12.4	7.8	23.2	7.3	12.0	11.6	21.6	10.1	9.3	3.7	11.3	7.6
	Median (MD)	48.8	32.8	39.0	43.2	78.1	41.8	40.6	42.6					34.5	28.1	60.7	32.3	46.0	34.8	35.2	42.4	49.4	39.4	29.3	51.7	58.9	35.1	33.2	43.8
	Min	47.2	25.8	27.0	25.0	50.7	27.2	26.3	39.9					28.8	13.8	27.3	25.0	31.0	18.4	30.2	30.3	22.3	17.7	15.3	24.3	40.1	26.8	6.8	36.0
	Max	80.8	48.0	97.0	46.8	82.0	44.8	78.6	44.8					44.7	39.2	84.0	52.8	61.3	39.9	86.0	53.0	71.6	57.3	86.0	57.8	70.2	40.6	41.8	58.4
	SEM	6.3	3.2	7.4	0.8	7.1	3.3	7.4	4.4					2.2	3.3	7.4	4.4	4.7	3.0	8.7	2.8	2.4	2.4	4.4	2.1	2.7	1.1	3.3	2.2

Italics mark the existing significant difference between two values

#### T2 (0–1 h)

At t2, mean parameter values of both, free and pedicled flaps, revealed similar in comparison between free and pedicled flaps with no significant differences (StO_2_, *p* = 0.75^(^*^)^; NIR, *p* = 0.36^(^*^)^; THI, *p* = 0.19^($)^; TWI, *p* = 1.0^($)^) (Table 1).

#### T3 (4–8 h)

For the period t3 (4 to 8 h after flap inset), parameter means were as follows: FF-StO_2_ (*m* 37.9 ± 17.0%); FF-NIR (*m* 35.5 ± 13.2); FF-THI (*m* 41.5 ± 29.3); FF-TWI (*m* 44.0 ± 15.7), whereby no data were available for PF.

#### T4 (8–12 h)

HSI measurements of t4 (8 to 12 h following flap inset) revealed no significant differences regarding perfusion parameters between free and pedicled flaps (StO_2_, *p* = 0.15^(^*^)^; NIR, *p* = 0.19^(^*^)^; THI, *p* = 0.57^($)^; TWI, *p* = 0.28^($)^) (Table 1).

#### T5 (12–24 h)

For HSI measurements between 12 and 24 h (t5), no significant differences of StO_2_, *p* = 0.76^(^*^)^; NIR, *p* = 0.56^(^*^)^; THI, *p* = 0.47^($)^; and TWI, *p* = 0.65^($)^ revealed when comparing free and pedicled flaps (Table 1).

#### T6 (24–48 h)

For the period between 24 and 48 h following flap inset (t6), there were no significant differences between StO_2_, *p* = 0.39^(^*^)^; NIR, *p* = 0.37^(^*^)^; THI, *p* = 0.11^($)^; and TWI, *p* = 0.30^($)^, when comparing free and pedicled flaps (Table 1).

#### T7 (> 48 h)

Regarding HSI measurements > 48 h following flap inset (t7), mean FF-THI (53.7 ± 16.7) was significantly increased compared with PF-THI (29.0 ± 11.3) (*p* = 0.00^($)^). However, StO_2_, NIR, and TWI were insignificantly different (StO_2_, *p* = 0.63^(^*^)^; NIR, *p* = 0.50^(^*^)^; TWI, *p* = 0.62^($)^) when comparing FF and PF (Table 1).

### Group-internal comparison of HSI parameters (StO_2_, NRI perfusion, THI, and TWI) at different time points (t1–t7) for free flaps

#### StO_2_

There was no significant difference between t1 and t2. In contrast, the tissue-oxygen-saturation (StO_2_) was significantly different between t1 and t3 (*p* = 0.00^(^*^)^), t1 and t4 (*p* = 0.00^(^*^)^), t1 and t5 (*p* = 0.00^(^*^)^), t1 and t6 (*p* = 0.00^(^*^)^), t1 and t7 (*p* = 0.00^(^*^)^), t2 and t3 (*p* = 0.01^(^*^)^), t2 and t4 (*p* = 0.02^(^*^)^), t2 and t5 (*p* = 0.001^(^*^)^), t2 and t6 (*p* = 0.004^(^*^)^), t2 and t7 (*p* = 0.015^(^*^)^), t3 and t5 (0.015^(^*^)^), and t3 and t7 (0.002^(^*^)^) as well as between t4 and t7 (0.023^(^*^)^). The other values are listed in Appendix 2a.

#### NIR perfusion

In the deeper tissue layers, the values were significantly different between the intervals t2 and t5 (*p* = 0.05^(^*^)^) as well as between t5 and t7 (*p* = 0.013^(^*^)^). There was no difference between the other measurement times for NRI perfusion (Table 6).

#### THI

The Tissue Haemoglobin Index (THI) was significantly increased at t4 compared with t2 (*p* = 0.049^($)^) as well as between t4 and t6 (*p* = 0.37^($)^). At the other time points, there was no significant difference (Table 6).

#### TWI

For the TWI, significant differences were found between t1 and t5 (*p* = 0.021^($)^), t1 and t6 (*p* = 0.00^($)^), t1 and t7 (*p* = 0.00^($)^), t3 and t7 (*p* = 0.036^($)^), and t4 and t7 (*p* = 0.035^($)^), and between t5 and t7 (*p* = 0.036^($)^). At the other time points, there was no significant difference (Table 6).

### Group-internal comparison of HSI parameters (StO_2_, NRI perfusion, THI, and TWI) at different time points (t1–t7) for pedicled flaps

#### StO_2_

Tissue-oxygen-saturation (StO_2_) in pedicled flaps was significantly different between t1 and t4 (*p* = 0.006^(^*^)^), t2 and t4 (*p* = 0.000^(^*^)^), t2 and t5 (*p* = 0.004^(^*^)^), t2 and t6 (*p* = 0.001^(^*^)^), t4 and t6 (*p* = 0.022^(^*^)^), and t4 and t7 (*p* = 0.001^(^*^)^) as well as between t6 and t7 (*p* = 0.044^(^*^)^). The other values are listed in Appendix 2b.

#### NIR perfusion

NIR perfusion showed significant differences over time between t4 and t6 (*p* = 0.015^(^*^)^). The other values are listed in Appendix 2b.

#### THI

The Tissue Haemoglobin Index (THI) was significantly increased at t4 compared with t7 (*p* = 0.007^($)^). The other values are listed in Appendix 2b.

#### TWI

No significant differences were revealed for the tissue water index (Table 6).

#### Cases with surgical re-entry

In overall 3/22 patients with FF reconstruction, surgical re-exploration was performed in consequence of flap deterioration whereby the salvage rate was 33% (1/3). HSI measurements conducted prior to = after flap inset^(pre-revision)^ and after revision^(post-revision)^ presented as follows:

##### Case 1^(OF-f)^: Re-exploration was performed 36 h after flap inset

HSI measurements^(pre-revision)^ showed a decline of StO_2_ (− 17%) and NIR (− 7) as well as an increase of the THI (+ 41.5) and TWI (+ 11.5) from baseline (t1) to hour 36 (t6). HSI measurements^(post-revision)^ were consistent with the further clinical course of an impaired perfusion due to venous congestion (bluish colour, immediate capillary refill, and deep red bleeding on puncture) leading to flap loss and explantation (StO_2_ − 6.1%^(t6/hour 40)^ to 20.9%^(t7/hour 58)^, NIR + 7.6^(t6/hour 40)^ to − 0.5^(t7/hour 67)^, THI − 6.6^(t6/hour 40)^ to + 17.8^(t7/hour 67)^, TWI − 3.2^(t6/hour 40)^ to − 7.3^(t7/hour 67)^). Detailed information is shown in Table 2.

**Table 2:** Development of HSI parameters from baseline (t1) to postsurgical measurement time points following re-exploration

	Presurgical			Postsurgical	
	t1	t6/hour 36		t7/hour 40	t7/hour 67
StO_2_ (%)	59.4	42.4	Re-entry	36.3	20.2
NIR	30.2	23.2		30.8	22.7
THI	4.4	45.9		39.3	63.7
TWI	28.7	40.2		36.3	32.9

##### Case 2^(RF-f)^: Re-exploration was performed 40 h after flap inset

HSI measurements^(pre-revision)^ revealed a decline of StO_2_ (− 38.7%), NIR (− 26.1), and THI (− 16.3) as well as an increase of the TWI (+ 15.3) from baseline (t1) to hour 40 (t6). These findings were in accordance with the clinical assessment likely to an impaired perfusion caused by an arterial occlusion (sudden pale colour, prolonged capillary refill time (> 5 s), absence of bleeding on needle puncture). After re-exploration, perfusion parameters increased continuously over time (Table 3).

**Table 3:** Development of HSI parameters from baseline (t1) to postsurgical measurement time points following re-exploration

	Presurgical			Postsurgical		
	t1	t6/hour 40		t7/hour 46	t7/hour 47	t7/hour 67
StO_2_ (%)	67.4	28.7	Re-entry	55.7	58.2	57.0
NIR	43.8	31.7		17.7	37.0	50.0
THI	48.0	33.3		35.7	55.4	76.5
TWI	32.4	47.7		34.7	47.0	46.0

##### Case 3^(RF-f)^: Take back and re-exploration were done 5 and 61 h following flap inset

HSI measurements^(pre-revision)^ revealed a decrease of StO_2_ (− 30.9%), NIR (− 7.5), and TWI (− 10.3) as well as an increase of the THI (+ 56) from baseline (t1) to hour 5 (t3). Hence, HSI measurements were in accordance with the clinical bedside assessment showing signs of a perfusion disorder (bluish colour, immediate capillary refill, and deep red bleeding on puncture), characteristic for a venous congestion. After the first take back with following revision at hour 5, flap perfusion initially increased until hour 14. However, flap perfusion again decreased within the further course leading to another take back, re-exploration of the cervical anastomosis, and final flap explantation in consequence of arterial and venous occlusion (no reflow phenomenon) at hour 61 (Table 4).

**Table 4:** Development of HSI parameters from baseline (t1) to postsurgical measurement time points following re-exploration

	Presurgical			Postsurgical			Presurgical
	t1	t3/hour 5		t4/hour 9	t5/hour 14		t7/hour 61
StO_2_ (%)	62.9	32.0	Re-entry	36.5	46.4	Re-entry	33.0
NIR	29.0	21.5		16.8	40.0		31.2
THI	38.5	95.3		95.5	97.0		77.1
TWI	29.3	19.0		15.0	26.6		30.4

## Discussion

Based on the present results, this study demonstrated the successful application of HSI for perioperative monitoring following free and pedicled flap transplantation. In addition, the findings provide an interpretation aid for clinicians to simplify postoperative flap assessment. Regarding HSI measurements immediately after flap inset (t1), direct perfusion parameter (StO_2_) revealed significantly (StO_2_, *p* = 0.05^(^*^)^) lower in pedicled flaps (PF-StO_2_, 56.2 ± 14.2%) when compared with free flaps (FF-StO_2_, 70.3 ± 14.8%). This could be caused by an increased blood flow as a result of iatrogenic denervation during vascular deposition with subsequent decrease in vascular resistance [15, 16]. Over time, flap perfusion decreased in both, free and pedicled flaps, with lowest values as follows: FF-StO_2_^(t3)^ 37.8%, FF-NIR^(t5)^ 29.7/100; PF-StO_2_^(t4)^ 35.8%; and PF-NIR^(t4)^ 27.6/100. In the further course, the perfusion increased in both flap types; however, neither StO_2_ nor NIR reached intraoperative levels (t1), which is in line with the findings of a former animal study presented by this study group [9].

With a correct true-positive detection rate of 3/3 impaired free flaps, the sensitivity of HSI imaging in this study was 100%. However, including two events of venous thrombus (cases 1 and 3) and one arterial occlusion (case 2), the salvage rate was only 33% (1/3), whereby perfusion disorders became clinically apparent 36 (case 1), 40 (case 2), and 5 h (case 3) following flap inset, representing—analogue to the literature—the critical time frame of 24 to 72 h for complications [2, 17, 18]. On closer examination of the hyperspectral parameters, signs of a disturbed perfusion could already be seen hours before the actual take back and revision of anastomosis. Case 1: By showing a decrease of superficial tissue oxygenation (StO_2_, 59.4 to 42.5% (− 17%)) and deep perfusion (NIR, 30.2 to 23.2 (− 7) in combination with an increase of THI (4.4 to 45.9 (+ 41.5) and TWI (28.7 to 40.2 (+ 11.5), HSI analysis reliably confirmed the clinical findings, characteristic for a venous congestion [9] and representing the basis for revision at hour 36. After surgical re-exploration and revision of the venous anastomosis, flap perfusion however remained poor with StO_2_ (21.5%^(t7/hour 58)^), NIR (22.7^(t7/hour 67)^), THI (63.7^(t7/hour 67)^), and TWI (32.9^(t7/hour 67)^), leading to a complete flap failure with subsequent flap explantation. Already at the time of measurement after 15 and 20 h, a constant decrease in superficial (StO_2_) and deep perfusion (NIR) was observed. This was additionally accompanied by an increase in the surrogate parameters THI and TWI. Thus, the hyperspectral analysis showed clear signs of incipient venous congestion 36 h prior to clinical diagnosis. As edema formation in soft tissue could be either based on renal dysfunction or impaired venous drainage, the TWI (Tissue Water Index) is a surrogate parameter providing information about the tissue water content [9], typically increased early (see case 1) from venous congestion and extravascular fluid leakage or after ischemia-induced endothelial death with a time lag. Regarding case 2, clinical signs of flap deterioration (sudden pale colour, prolonged capillary refill time, absence of bleeding on needle puncture) became clinically obvious 40 h after flap inset, coming along with a change of HSI parameters (StO_2_, 67.4 to 28.7 (− 38.7%); NIR, 43.8 to 17.7 (− 26.1); THI, 48.0 to 31.7 (− 16.3); and TWI, 32.4 to 47.7 (+ 15.3)). After re-exploration and revision of the arterial anastomosis within 1 h, flap perfusion improved clinically (pink colour, capillary refill after 2 to 3 s) which was also confirmed by HSI measurements during the further course (61.5%^(t7/hour 59)^), NIR (58.5^(t7/hour 59)^); THI (67.5^(t7/hour 59)^), and TWI (55.5^(t7/hour 59)^). According to the HSI analysis, however, the perfusion parameters already showed a decrease from hour 25 to 27 (StO_2_, − 19% and NIR, − 20) and thus gave an indication on a compensated course 13 h prior to clinical diagnosis. Together with the parallel value progression of THI, this appears to be consistent with the hyperspectral signature pattern characteristic for arterial perfusion disorders. In case 3, flap perfusion declined clinically 5 h after flap inset, showing signs characteristic for venous congestion (bluish colour, immediate capillary refill, and deep red bleeding on puncture). This again was reflected in a typical value curve of the hyperspectral parameters (StO_2_ 76.5 to 32.0% (− 44.5%)), NIR (44.8 to 21.5 (− 23.3), THI (91.0 to 95.3 (+ 4.3)). After partial externalisation of the FF for tension relief and initial increase of direct perfusion parameters (StO_2_ and NIR), flap perfusion deteriorated again clinically 61 h after flap inset, which was also reflected in the HSI measurements (StO_2_ down to 33.0%^(t7/hour 61)^; NIR, down to 31.2^(t7/hour 61)^; THI, down to 77.1^(t7/hour 61)^; TWI, 30.4^(t7/hour 61)^) leading to surgical re-exploration and subsequent flap explantation due to a persisting arterial occlusion. Clinical evaluation was always in line with HSI and performed by an experienced surgeon (PWK). Taking THI into account, however, a critical increase is already evident after 1.5 h, giving an early (3.5 h prior to clinical diagnosis) indication of venous congestion. In addition, prior to the second revision, a decrease of superficial (StO_2_) and deep perfusion (NIR) was already evident 25 h after flap inset, thus 36 h prior to the clinical diagnosis. However, this conclusion can only be drawn with regard to the further course, since parameters were within an acceptable range. In accordance to the course of case 1, usually venous congestion causes a gradual decline in flow values that often lasts for hours, while arterial occlusion leads to an abrupt decline [19]. In comparison with pulse oximetry as another noninvasive technique to assess tissue oxygenation, the HSI provides discriminable monitoring of both, arterial and venous oxygenation [20]. Yoshino et al. was unable to distinguish between arterial and venous problems when monitoring 37 intraoral free flaps with a laser Doppler flowmeter [21]. In contrast, Hölzle et al. described the successful application of O_2_C (oxygen-to-see, LEA-Medizintechnik GmbH, Gießen, Germany), a device combination of a laser Doppler flowmeter and a tissue spectrometer, for free flap monitoring and early recognition of flap failure [22]. For measurements, a probe has to be continuously in contact with the flap surface, giving quantitative information about a limited area only. Flap monitoring with laser-induced fluorescence of indocyanine green (ICG) was described as extremely sensitive and able to detect even partial flap necrosis [23]. However, monitoring requires expertise and the injection of fluorescein carries the risk for anaphylactic reactions; thus, ICG is contraindicated in patients with iodine allergy [1, 24]. Besides many other methods for flap monitoring, e.g. confocal microscopy, pH measurement, Sidestream Dark Field Imaging, temperature measurement, and the usage of implantable Doppler probes, the HSI works completely contactless conveying much more spectral information than truecolour RGB (red, green, blue) or other multispectral data systems. In addition, due to image acquisition, HSI by TIVITA™ provides both spectral and spatial information about the respective area of interest (visible flap surface). However, it should be noted that ambient light conditions always slightly differ between the measurements having potential impact on the parameters’ values. One further disadvantage of this HSI system is the housing size of the measuring unit allowing only limited illumination of the posterior sections of the oral cavity (Table 5). The examination quality of free muscle flaps with split skin coverage is also questionable. Current investigations of the authors showed a high dependency on the quality of the split-skin covering, since subcutaneous seroma accumulations due to an insufficient split-skin-fit leads to a falsification of values. Former studies have already described the successful usage of the HSI whereas Köhler et al. described HSI as a valuable method for evaluating ischaemic conditioning effects of the gastric conduit during oesophagectomy [25]. To the authors’ knowledge, this is the first study assessing the feasibility of HSI to monitor flap perfusion following reconstructive operations in the field of oral and maxillofacial surgery. The data shown here demonstrate for the first time the successful clinical use of hyperspectral technology for transplant monitoring and underline the preclinical results of this research group. Conclusive statements on the exact definition of threshold values cannot be made on the basis of the available results, but they provide an important contribution to further investigations. Based on the number of cases, particularly those needing subsequent re-exploration, the present results have to be classified as of descriptive nature. Hence, further clinical studies must be conducted in order to generate more data that supports the development of threshold values for therapy recommendations. In this context, great potential may arises from a combination of deep learning technologies and HSI [26]. Another research approach of the working group is the fusion of artificial intelligence (e.g. neuronal network approaches) and hyperspectral technology. In the foreseeable future, this would eliminate the need for result interpretation by the user and thus enable safe clinical application by the less experienced physician or even nursing staff in order to save resources.

## Conclusion

Hyperspectral imaging permits early and objective detection of flap failure prior to clinical assessment. Based on the present results, the necessity for anastomosis revision arose in cases of StO_2_ values < 45% and NIR values below 25/100. A gradual decline of StO_2_ and NIR in combination with an increase of THI indicated venous congestion. In contrast, a sudden decline of StO_2_, NIR, and THI was shown. A multi-centre approach is needed to establish threshold values, given the generally low number of revisions.

### Electronic supplementary material

ESM 1 (DOC 274 kb)

ESM 2 (DOC 200 kb)

## Appendix

**Table 5:** (A) shows the construction of the measuring unit with (1) the measuring head with lens, halogen lamps, and own control unit attached to a telescopic arm. This is attached to a mobile trolley with control computer, operating elements (keyboard and mouse), and screen (2). (B) shows the user interface of the software with selection of the body region to be measured (here the face). (C) shows a live measurement of a person (here a medical colleague). (D) shows the alignment of the measuring unit with focus light switched on with projection on the right cheek

Dependant variable	Measurement time point (interval)	Measurement interval	Significance (*p* =)
StO_2_ (%)	t1	t2	.810^(^*^)^
		t3	*.000*^*(*^***^*)*^
		t4	*.000*^*(*^***^*)*^
		t5	*.000*^*(*^***^*)*^
		t6	*.000*^*(*^***^*)*^
		t7	*.000*^*(*^***^*)*^
	t2	t1	.810^(^*^)^
		t3	*.001*^*(*^***^*)*^
		t4	*.002*^*(*^***^*)*^
		t5	*.012*^*(*^***^*)*^
		t6	*.004*^*(*^***^*)*^
		t7	*.015*^*(*^***^*)*^
	t3	t1	*.000*^*(*^***^*)*^
		t2	*.001*^*(*^***^*)*^
		t4	.560^(^*^)^
		t5	*.015*^*(*^***^*)*^
		t6	.063^(^*^)^
		t7	*.002*^*(*^***^*)*^
	t4	t1	*.000*^*(*^***^*)*^
		t2	*.002*^*(*^***^*)*^
		t3	.560^(^*^)^
		t5	.085^(^*^)^
		t6	.280^(^*^)^
		t7	*.023*^*(*^***^*)*^
	t5	t1	*.000*^*(*^***^*)*^
		t2	*.012*^*(*^***^*)*^
		t3	*.015*^*(*^***^*)*^
		t4	*.085*^*(*^***^*)*^
		t6	.317^(^*^)^
		t7	.647^(^*^)^
	t6	t1	*.000*^*(*^***^*)*^
		t2	*.004*^*(*^***^*)*^
		t3	*.063*^*(*^***^*)*^
		t4	.280^(^*^)^
		t5	.317^(^*^)^
		t7	*.077*^*(*^***^*)*^
	t7	t1	*.000*^*(*^***^*)*^
		t2	*.015*^*(*^***^*)*^
		t3	*.002*^*(*^***^*)*^
		t4	*.023*^*(*^***^*)*^
		t5	.647^(^*^)^
		t6	.077^(^*^)^
NIR perfusion	t1	t2	.152^(^*^)^
		t3	.780^(^*^)^
		t4	.496^(^*^)^
		t5	.188^(^*^)^
		t6	.724^(^*^)^
		t7	.440^(^*^)^
	t2	t1	.152^(^*^)^
		t3	.123^(^*^)^
		t4	.087^(^*^)^
		t5	**.050**^(^*^)^
		t6	.111^(^*^)^
		t7	.231^(^*^)^
	t3	t1	.780^(^*^)^
		t2	.123^(^*^)^
		t4	.691^(^*^)^
		5	.336^(^*^)^
		t6	.995^(^*^)^
		t7	.289^(^*^)^
	t4	t1	.496^(^*^)^
		t2	.087^(^*^)^
		t3	.691^(^*^)^
		t5	.622^(^*^)^
		t6	.636^(^*^)^
		t7	.136^(^*^)^
	t5	t1	.188^(^*^)^
		t2	*.050*^*(*^***^*)*^
		t3	.336^(^*^)^
		t4	.622^(^*^)^
		t6	.216^(^*^)^
		t7	*.013*^*(*^***^*)*^
	t6	t1	.724^(^*^)^
		2	.111^(^*^)^
		t3	.995^(^*^)^
		t4	.636^(^*^)^
		t5	.216^(^*^)^
		7	.133^(^*^)^
	t7	t1	.440^(^*^)^
		t2	.231^(^*^)^
		t3	.289^(^*^)^
		t4	.136^(^*^)^
		t5	*.013*^*(*^***^*)*^
		t6	.133^(^*^)^
THI	t1	t2	.192^($)^
		t3	.715^($)^
		t4	.108^($)^
		t5	.434^($)^
		t6	.545^($)^
		t7	.485^($)^
	t2	t1	.192^($)^
		t3	.255^($)^
		t4	.046^($)^
		t5	.104^($)^
		t6	.261^($)^
		t7	.113^($)^
	t3	t1	.715^($)^
		t2	.255^($)^
		t4	.059^($)^
		t5	.257^($)^
		t6	.887^($)^
		t7	.276^($)^
	t4	t1	.108^($)^
		t2	.046^($)^
		t3	.059^($)^
		t5	.325^($)^
		t6	.016^($)^
		t7	.210^($)^
	t5	t1	.434^($)^
		t2	.104^($)^
		t3	.257^($)^
		t4	.325^($)^
		t6	.103^($)^
		t7	.839^($)^
	t6	t1	.545^($)^
		t2	.261^($)^
		t3	.887^($)^
		t4	.016^($)^
		t5	.103^($)^
		t7	.082^($)^
	t7	t1	.485^($)^
		t2	.113^($)^
		t3	.276^($)^
		t4	.210^($)^
		t5	.839^($)^
		t6	.082^($)^
TWI	t1	t2	.467^($)^
		t3	.213^($)^
		t4	.687^($)^
		t5	.049^($)^
		t6	.000^($)^
		t7	.000^($)^
	t2	t1	.467^($)^
		t3	.854^($)^
		t4	.590^($)^
		t5	.965^($)^
		t6	.604^($)^
		t7	.386^($)^
	t3	t1	.213^($)^
		t2	.854^($)^
		t4	.437^($)^
		t5	.576^($)^
		t6	.062^($)^
		t7	.005^($)^
	t4	t1	.687^($)^
		t2	.590^($)^
		t3	.437^($)^
		t5	.165^($)^
		t6	.007^($)^
		t7	.000^($)^
	t5	t1	.049^($)^
		t2	.965^($)^
		t3	.576^($)^
		t4	.165^($)^
		t6	.144^($)^
		t7	.011^($)^
	t6	t1	.000^($)^
		t2	.604^($)^
		t3	.062^($)^
		t4	.007^($)^
		t5	.144^($)^
		t7	.193^($)^
	t7	t1	.000^($)^
		t2	.386^($)^
		t3	.005^($)^
		t4	.000^($)^
		t5	.011^($)^
		t6	.193^($)^

Italics mark the existing significant difference between two values

**Table 6:** Tabular listing of the significance tests within the groups free (a) and pedicled (b) flaps between the different measurement intervals. Here ^(^*^)^ stands for the statistical significance test with the Students *t* test, and ($) for the test with Mann-Whitney *U* test

Dependant variable	Measurement timepoint (interval)	Measurement interval	Significance (*p* =)
StO_2_ (%)	t1	t2	.085^(^*^)^
		t4	*.006*^*(*^***^*)*^
		t5	.260^(^*^)^
		t6	.182^(^*^)^
		t7	.902^(^*^)^
	t2	t1	.085^(^*^)^
		t4	*.000*^*(*^***^*)*^
		t5	*.004*^*(*^***^*)*^
		t6	*.001*^*(*^***^*)*^
		t7	.055^(^*^)^
	t4	t1	*.006*^*(*^***^*)*^
		t2	*.000*^*(*^***^*)*^
		t5	.058^(^*^)^
		t6	*.022*^*(*^***^*)*^
		t7	*.001*^*(*^***^*)*^
	t5	t1	.260^(^*^)^
		t2	*.004*^*(*^***^*)*^
		t4	.058^(^*^)^
		t6	.998^(^*^)^
		t7	.130^(^*^)^
	t6	t1	.182^(^*^)^
		t2	*.001*^*(*^***^*)*^
		t4	*.022*^*(*^***^*)*^
		t5	.998^(^*^)^
		t7	*.044*^*(*^***^*)*^
	t7	t1	.902^(^*^)^
		t2	.055^(^*^)^
		t4	*.001*^*(*^***^*)*^
		t5	.130^(^*^)^
		t6	*.044*^*(*^***^*)*^
NIR_mean	t1	t2	.633^(^*^)^
		t4	.150^(^*^)^
		t5	.472^(^*^)^
		t6	.590^(^*^)^
		t7	.861^(^*^)^
	t2	t1	.633^(^*^)^
		t4	.055^(^*^)^
		t5	.219^(^*^)^
		t6	.940^(^*^)^
		t7	.459^(^*^)^
	t4	t1	.150^(^*^)^
		t2	.055^(^*^)^
		t5	.412^(^*^)^
		t6	*.015*^*(*^***^*)*^
		t7	.120^(^*^)^
	t5	t1	.472^(^*^)^
		t2	.219^(^*^)^
		t4	.412^(^*^)^
		t6	.114^(^*^)^
		t7	.490^(^*^)^
	t6	t1	.590^(^*^)^
		t2	.940^(^*^)^
		t4	*.015*^*(*^***^*)*^
		t5	.114^(^*^)^
		t7	.313^(^*^)^
	t7	t1	.861^(^*^)^
		t2	.459^(^*^)^
		t4	.120^(^*^)^
		t5	.490^(^*^)^
		t6	.313^(^*^)^
THI mean	t1	t2	.803^($)^
		t4	.525^($)^
		t5	.999^($)^
		t6	.205^($)^
		t7	.053^($)^
	t2	t1	.803^($)^
		t4	.371^($)^
		t5	.786^($)^
		t6	.341^($)^
		t7	.099^($)^
	t4	t1	.525^($)^
		t2	.371^($)^
		t5	.490^($)^
		t6	.057^($)^
		t7	*.007*^*($)*^
	t5	t1	.999^($)^
		t2	.786^($)^
		t4	.490^($)^
		t6	.147^($)^
		t7	0.151^($)^
	t6	t1	.205^($)^
		t2	.341^($)^
		t4	.030^($)^
		t5	.147^($)^
		t7	.238^($)^
	t7	t1	.053^($)^
		t2	.099^($)^
		t4	*.007*^*($)*^
		t5	.031^($)^
		t6	.238^($)^
TWI mean	t1	t2	.715^($)^
		t4	.451^($)^
		t5	.708^($)^
		t6	.098^($)^
		t7	.143^($)^
	t2	t1	.715^($)^
		t4	.258^($)^
		t5	.984^($)^
		t6	.232^($)^
		t7	.298^($)^
	t4	t1	.451^($)^
		t2	.258^($)^
		t5	.227^($)^
		t6	.062^($)^
		t7	.053^($)^
	t5	t1	.708^($)^
		t2	.984^($)^
		t4	.227^($)^
		t6	.164^($)^
		t7	.235^($)^
	t6	t1	.098^($)^
		t2	.232^($)^
		t4	.062^($)^
		t5	.164^($)^
		t7	.921^($)^
	t7	t1	.143^($)^
		t2	.298^($)^
		t4	.053^($)^
		t5	.235^($)^
		t6	.921^($)^

Italics mark the existing significant difference between two values
